# Caspase inhibitors affect the kinetics and dimensions of tracheary elements in xylogenic Zinnia (*Zinnia elegans*) cell cultures

**DOI:** 10.1186/1471-2229-10-162

**Published:** 2010-08-06

**Authors:** Peter Twumasi, Elena T Iakimova, Tian Qian, Wim van Ieperen, Jan HN Schel, Anne Mie C Emons, Olaf van Kooten, Ernst J Woltering

**Affiliations:** 1 Wageningen University, Plant Sciences Group, Horticultural Supply Chains, P.O. Box 630, 6700 AP Wageningen, The Netherlands; 2 Wageningen University, Food and Biobased Research, PO Box 17, 6700 AA Wageningen, The Netherlands; 3 Department of Biochemistry and Biotechnology, Kwame Nkrumah University of Science and Technology (KNUST), Kumasi, Ghana; 4 Institute of Ornamental Plants, 1222 Negovan, Sofia, Bulgaria; 5 Wageningen University, Laboratory of Plant Cell Biology, P.O. Box 633, 6700 AP Wageningen, The Netherlands

## Abstract

**Background:**

The xylem vascular system is composed of fused dead, hollow cells called tracheary elements (TEs) that originate through trans-differentiation of root and shoot cambium cells. TEs undergo autolysis as they differentiate and mature. The final stage of the formation of TEs in plants is the death of the involved cells, a process showing some similarities to programmed cell death (PCD) in animal systems. Plant proteases with functional similarity to proteases involved in mammalian apoptotic cell death (caspases) are suggested as an integral part of the core mechanism of most PCD responses in plants, but participation of plant caspase-like proteases in TE PCD has not yet been documented.

**Results:**

Confocal microscopic images revealed the consecutive stages of TE formation in Zinnia cells during trans-differentiation. Application of the caspase inhibitors Z-Asp-CH2-DCB, Ac-YVAD-CMK and Ac-DEVD-CHO affected the kinetics of formation and the dimensions of the TEs resulting in a significant delay of TE formation, production of larger TEs and in elimination of the 'two-wave' pattern of TE production. DNA breakdown and appearance of TUNEL-positive nuclei was observed in xylogenic cultures and this was suppressed in the presence of caspase inhibitors.

**Conclusions:**

To the best of our knowledge this is the first report showing that caspase inhibitors can modulate the process of trans-differentiation in Zinnia xylogenic cell cultures. As caspase inhibitors are closely associated with cell death inhibition in a variety of plant systems, this suggests that the altered TE formation results from suppression of PCD. The findings presented here are a first step towards the use of appropriate PCD signalling modulators or related molecular genetic strategies to improve the hydraulic properties of xylem vessels in favour of the quality and shelf life of plants or plant parts.

## Background

Maintenance and structuring of tissues and organs, homeostasis and defence in biological organisms are controlled by well coordinated and active cellular processes of death and survival. Programmed cell death (PCD) is a cell suicide genetically programmed, developmentally associated and environmentally stimulated mechanism and has been found throughout animal and plant kingdoms [1-6]. PCD is aimed at establishing functional structures, at removal of cells that are harmful or no longer needed, and it plays a prominent role in resistance and adaptation to abiotic and biotic insults. In mammalian cells, a type of PCD commonly referred to as apoptosis, is mediated by cysteinyl-aspartic proteases (caspases), involving initiator caspases and downstream executioner caspases. This results in a typical phenotype comprised of cell shrinkage, chromatin condensation, inter nucleosomal DNA cleavage and, cellular fragmentation into apoptotic bodies, that are digested by macrophages [1,7,8]. Caspases show a high degree of specificity with an absolute requirement for cleavage adjacent an aspartic acid residue and a recognition sequence of at least four amino acids N-terminal to this cleavage site [9]. No structural homologues of animal caspases exist in plants, but caspase peptide inhibitors designed to inhibit human caspases have been proven as potent inhibitors of PCD in plant systems as well. This indicates the existence in plants of proteases with substrate specificity and functional similarity to animal caspases [10-15]. Different types of metacaspases, saspases and vacuolar processing enzymes may be involved in specific cases of plant PCD [16-21]. Most of the morphological and biochemical features associated with animal apoptosis have also been identified in dying plant cells [22-25]. Other types of cell death, such as autophagy and non-lysosomal cell death are described to occur as forms of plant PCD. A regulatory overlap between different plant PCD types is suggested to operate depending on the inducing stimuli and rapidity with which cell death is required [6,26].

In vascular plants, PCD is involved in embryogenesis, developmental processes, senescence, hypersensitive response to pathogen attacks and in the response to abiotic stress stimuli [3,5,19,27]. A typical example of developmentally regulated PCD is PCD that occurs at the final stage of cell differentiation during the formation of the xylem vascular system [2,28,29]. Xylem vessels (water conducting tubes) are composed of a number of fused vessel or tracheary elements (TEs) that are dead, hollow cells with patterned lignified cellulose secondary walls. TEs originate through differentiation of root and shoot pro-cambium and cambium cells [23] and undergo autolysis as they differentiate and mature [28].

The development of xylogenic Zinnia (*Zinnia elegans*) cell cultures, derived from leaf mesophyll cells, has revolutionized the understanding of the xylem differentiation process [30]. Three consecutive stages, each associated with specific physiological states of the cells, typical morphological features and expression of specific sets of genes have been described during the process of trans-differentiation of Zinnia cells. Stage I includes de-differentiation of mesophyll cells and acquisition of competence for re-differentiation; during stage II synthesis and deposition of secondary wall material occurs and, stage III involves a progression of the PCD process associated with (in this order) the formation of a large vacuole, rupture of the tonoplast, DNA fragmentation, disappearing of the nucleus, autolysis of cell content and formation of hollow dead TEs [28,31-34].

Involvement of PCD in the process of TE differentiation suggests that xylogenesis could be manipulated by controlling the initiation and the progress of PCD, which in turn may alter the anatomy of the formed TEs. Hence, as the xylem system architecture influences the hydraulic and mechanical properties of the plant, the use of appropriate PCD signalling modulators may lead to modified properties of the xylem vessels that may affect the quality of the plant or specific plant parts.

The differentiation process *in vitro *and *in planta *is regulated by endogenous and exogenous factors. Plant hormones (cytokinins and auxins) are required for trans-differentiation [35,36] and other signalling molecules and metabolic pathways (e.g. brassinosteroids, sulphated peptide hormone phytosulfokine, calcium, ROS, abscisic and jasmonic acids, nitric oxide, ethylene, MAP-kinases, pH and osmotic values) are described to be involved in the integration and control of TE formation [36-41]. In xylogenic Zinnia cell cultures, a portion of the cells do not differentiate but might serve as feeder cells to supply substrate facilitating the differentiation of the others [42,43]. The impact of cells differentiating into TE's on the differentiation of other neighbouring cells and *vice versa *is not yet clear. It is suggested that differentiating cells might send messenger molecules to the neighbouring cells to initiate or prevent xylogenesis. A non-classical type of arabinogalactan protein is suggested to fulfil a function of intercellular communicator, known as hypothetical xylogen [44].

On the basis of their expression patterns various serine and cysteine proteases and ribonucleases, have been demonstrated to be implicated in the control of xylem PCD [4,29,44-47]. Newly synthesized S1-nuclease (ZEN1) [48] has been shown to function in nuclear DNA degradation [49] during PCD of Zinnia tracheary elements. Two *Arabidopsis thaliana *proteases, xylem cysteine protease 1 (XCP1) and 2 (XCP2), have been identified to participate in micro-autolysis within the intact central vacuole before mega-autolysis is initiated by tonoplast rupture [50]. Other hydrolytic enzymes, such as RNases (ZRNase1) and cysteine proteases (ZCP4) [36] are also reported to accumulate in the vacuole of differentiating Zinnia cells and are released after vacuolar collapse [51].

Although caspase-like proteases are suggested an integral part of the core mechanism of most PCD responses in plants, their possible role in xylem vessel PCD has not been established [21]. Microarray analysis has shown upregulation of a metacaspase 9, *VPEα*, and xylem cysteine proteases during TE differentiation in *Arabidopsis *[33]. By immunohistochemistry and immunoelectron microscopy, caspase-3-like protease has been detected in developing tracheary elements in *Cucurbita moschata *[52], but no evidence has as yet been provided about the functional involvement of plant caspase-like proteases in TE development. The existing information based on pharmacological studies in Zinnia cell cultures suggests that caspase-like proteases do not play a role in TE formation [21,28,33,53].

In this study we have investigated the potency of specific peptide caspase inhibitors (the broad range irreversible caspase inhibitor Z-Asp-CH2-DCB, irreversible caspase 1 inhibitor Ac-YVAD-CMK and reversible caspase 3 inhibitor Ac-DEVD-CHO) to modulate the development of TEs in xylogenic Zinnia cell cultures. The application of caspase inhibitors affected the kinetics of formation and the final dimensions of the TEs, resulting in a significant delay of the time of TE formation and stimulation of the development of TEs with larger dimensions (length and area). DNA breakdown and appearance of TUNEL-positive nuclei associated with TE formation was inhibited in presence of caspase inhibitors. Confocal microscopic images revealed a significant delay but not a re-ordering of the consecutive stages of TE formation in caspase inhibitor treated cells. To the best of our knowledge this is the first report providing information that in xylogenic Zinnia cell cultures application of caspase inhibitors affects the process of trans-differentiation.

## Results

### Effect of caspase inhibitors on trans-differentiation

After first being introduced by Fukuda and Kommamine [30] the Zinnia cell culture has become a convenient model to study the process of xylogenesis *in vitro*. Three peptide caspase inhibitors, known to interfere with PCD events in mammalian systems and proven to be efficient cell death suppressors in a diversity of plant systems were tested for their potency to influence the trans-differentiation of leaf mesophyll cells into TEs in Zinnia suspension cell cultures. A cell showing secondary cell wall banding, whether dead or alive is considered a TE.

When different concentrations (1-1000 nM) of the caspase inhibitors were tested in non-induced cultures, cell viability after 5 days was generally higher than in the non-inhibitor treated cultures (data not shown). Concentrations higher than 1000 nM were found to be toxic. Therefore, in further experiments we routinely treated the cells with concentrations of up to 100 nM.

**Figure 1:**
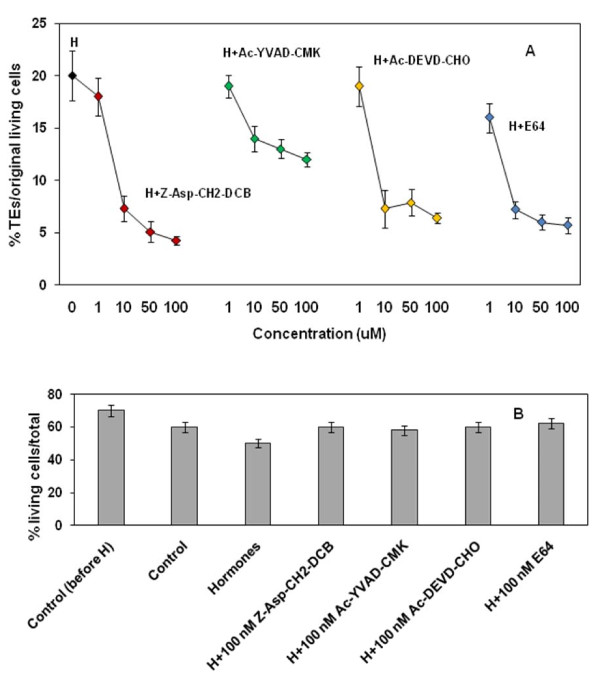
**Effect of caspase inhibitors and a cysteine protease inhibitor on TE formation in Zinnia cell cultures**. A. Effect of inhibitor concentrations on the percent of formed TEs; H - hormone induced culture; B. Effect of inhibitors on cell viability. The cells were treated with 1 mg/L NAA and 1 mg/L BA and combinations of these hormones with the broad spectrum caspase inhibitor Z-Asp-CH2-DCB, the irreversible caspase-1- inhibitor Ac-YVAD-CMK, the reversible caspase 3 inhibitor Ac-DEVD-CHO and the cysteine protease inhibitor E-64 at indicated concentrations. Control cell cultures were left untreated. Cell viability was scored 120 h after addition of the chemicals as a percentage of living cells to the total number of cells and the number of TEs was calculated as a percentage of formed TEs to the initial number of living cells. 0.002% FDA was used to stain the living cells and 0.0005% CFW was used to stain the secondary cell walls of the differentiated cells. Cell counting of three non overlapping microscopic fields per each sample was performed with a fluorescence microscope (Axiovert, Carl Zeiss, Darmstadt, Germany) at 100× magnification. Presented data are averages of at least three independent experiments. Error bars indicate SEM _(n-1)_.

Trans-differentiation was induced with 1 mg/L NAA and 1 mg/L BA. Cultures were simultaneously treated with hormones and 1, 10, 50 and 100 nM concentrations of the irreversible broad spectrum caspase inhibitor Z-Asp-CH2-DCB, the irreversible caspase 1 inhibitor Ac-YVAD-CMK and the reversible caspase 3 inhibitor Ac-DEVD-CHO. Within 5 days of treatment, approximately 20% of the initially living mesophyll cells trans-differentiated to form TEs in response to the plant hormones. The caspase inhibitors suppressed TE formation in a concentration dependent way (Fig. 1A). A remarkable 80% inhibition of trans-differentiation occurred after administration of Z-Asp-CH2-DCB. Lower, but substantial inhibition was detected at the application of 100 nM Ac-DEVD-CHO and Ac-YVAD-CMK (70 and 40%, respectively). The cell viability (% living cells of total) in hormone-induced cultures was about 10% lower than in the negative control (non-hormone treated) probably reflecting the increased number of TEs formed from living cells (Fig. 1B). In the presence of caspase inhibitors cell viability did not differ significantly from non-hormone control (Fig. 1B). These results indicate that plant proteases with substrate specificity comparable to animal caspases are involved in the trans-differentiation process in Zinnia xylogenic cell cultures.

The cell cultures were also exposed to treatments with irreversible cysteine protease inhibitor E-64 (concentration range 1-100 nM) (Fig. 1A) and the cysteine protease inhibitors IA (5 μM) and NEM (50 μM). TE generation was inhibited by about 75% in response to the addition of 100 nM E64 (Fig. 1A) and no trans-differentiation at all was detected at the addition of IA and NEM (data not shown). This shows that cysteine proteases other than caspase-like proteases are also involved in the trans-differentiation process.

### Effect of caspase inhibitor Z-Asp-CH2-DCB on DNA fragmentation

**Figure 2:**
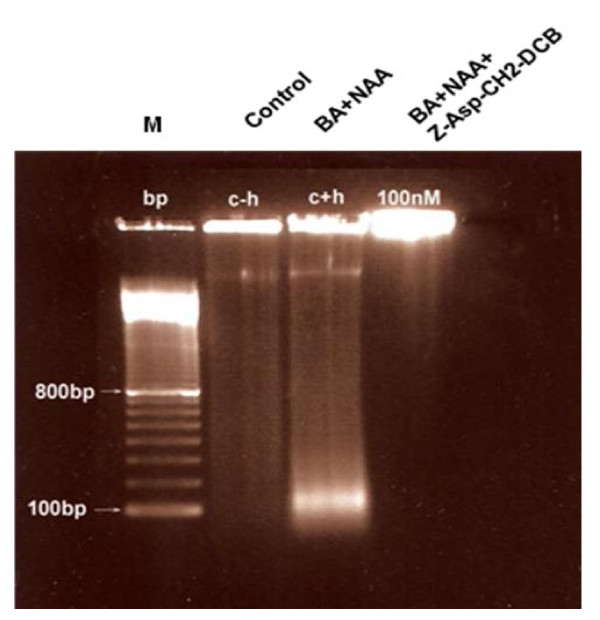
**Effect of Z-Asp-CH2-DCB on DNA fragmentation in xylogenic Zinnia cell cultures**. The cells were harvested 120 h after the induction of differentiation. DNA was isolated, separated and stained with ethidium bromide as described in *Material and Methods*. M, marker 1 kb; lane 1 (c - h), negative control of non-induced cells; lane 2 (c + h), 1 mg/L NAA + 1 mg/L BA; lane 3, 1 mg/L NAA + 1 mg/L BA + 100 nM of the of the irreversible broad spectrum caspase inhibitor Z-Asp-CH2-DCB.

DNA isolation and separation by agarose gel electrophoresis was performed to study whether the inhibition of TE differentiation in response to treatments with caspase inhibitors affects PCD -associated DNA degradation. DNA was collected 120 h after hormonal induction of xylogenesis. No DNA fragmentation was detected in control (non-hormone induced) cells (Fig. 2, lane 2). Oligonucleosomal DNA fragments of around 160-180 bp were clearly visible in xylogenic cell cultures (Fig. 2, lane 3). DNA fragmentation was greatly inhibited in the presence of 100 nM Z-Asp-CH2-DCB (Fig. 2, lane 4). These results indicate that indeed the TE trans-differentiation is associated with DNA fragmentation and that the caspase inhibitor Z-Asp-CH2-DCB efficiently prevents this phenomenon.

**Figure 3:**
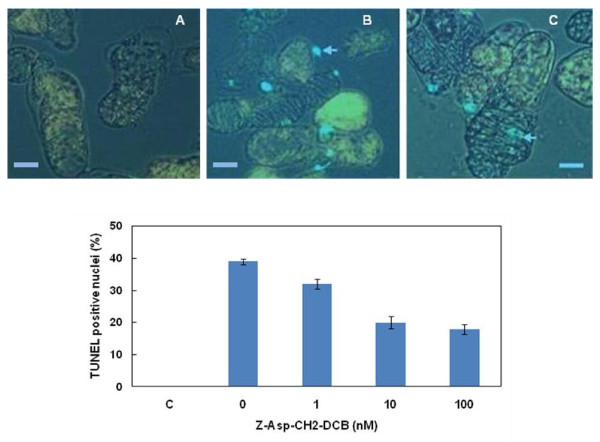
**TUNEL assay of condensed nuclei in Zinnia suspension cultured cells exposed to treatment with Z-Asp-CH2-DCB**. A. Negative control from a non-induced cultures; B. cells treated with 1 mg/L NAA and 1 mg/L BA; C. 1 mg/L NAA + 1 mg/L BA + 100 nM Z-Asp-CH2-DCB. Images were collected with a Nikon fluorescence microscope at 510 nm wavelength. Green fluorescent dots (arrowheads) indicate nuclei containing cleaved DNA. Scale bars (A, B, C) = 30 μm. D. Effect of Z-Asp-CH2-DCB on the amount of TUNEL positive nuclei (TUNEL scored as percent of cells with secondary wall) in which: C, negative control from non-inductive cultures; 0, cells treated with 1 mg/L NAA and 1 mg/L BA; the remaining variants are combinations of the hormones with 1, 10 or 100 nM Z-Asp-CH2-DCB. Error bars indicate SEM_(n-1)_.

To further investigate the DNA fragmentation, DNA double strand breaks were identified by TUNEL assay. TUNEL staining is a relatively early marker of DNA fragmentation and therefore we tested after 72 h of treatment, when first TEs are formed in hormone-treated cultures. Control cells (non-hormone treated), hormone treated cells and cells treated with hormones and different concentrations of Z-Asp-CH2-DCB were fixed and subjected to TUNEL assay. The intact nuclei containing fragmented DNA show fluorescence at 510 nm emission wave length (TUNEL-positive nuclei). No TUNEL-positive nuclei were detected in non-differentiating control cells (Fig. 3A). TUNEL-positive nuclei were visible in differentiating cells in hormone treated cultures (Fig. 3B). Approximately 50% reduction of the percentage of TUNEL positive nuclei was established after the addition of 10 or 100 nM Z-Asp-CH2-DCB (Fig. 3C, D). These observations on DNA breakdown and TUNEL staining were confirmed using different caspase inhibitors and in another Zinnia cultivar (Purple Prince) (data not shown). The observed internucleosomal DNA cleavage and the occurrence of TUNEL-positive nuclei, during trans-differentiation suggest that PCD is an integral part of the trans-differentiation process. Z-Asp-CH2-DCB, but also the other caspase inhibitors (data not shown) and the cysteine protease inhibitor E64 (data not shown) greatly suppressed DNA breakdown which suggests their involvement in PCD during trans-differentiation.

### Morphology of the TE forming cells

The morphological changes during TE formation were examined using confocal laser-scanning microscopy. Living, non-differentiated cells were distinguished from dead cells by fluorescein diacetate (FDA) staining. Nuclei in the dead cells were visualized with propidium iodide (PI). Cell wall thickenings in the differentiated cells were imaged by calcofluor white (CFW), specific for staining of cellulose cell wall depositions.

**Figure 4:**
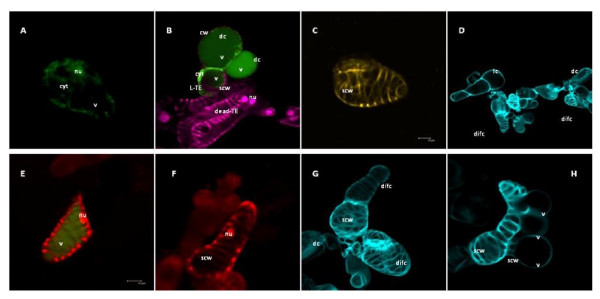
**Confocal images of Zinnia suspension cultured cells, representing the cell morphology during TEs differentiation**. A. FDA stained living cell - visible are diffuse nuclei, intact cytoplasm and intact vacuole; B. Double FDA-PI stained cells showing different stages of vacuole rupture: living TE with intact vacuole, preserved cytoplasm and single SCW, lacking a "dead" nucleus (the lower cell), dead cell - FDA is partially penetrated in the vacuole, but the cellular organelles are still preserved (the upper larger cell); dead cell with ruptured vacuole and lacking FDA stained cytoplasm (right), dead TEs with "dead" nuclei (underneath the living TE) - PI fluorescence is visualised in pink; C. Fully differentiated TE - autofluorescence from the lignified secondary cell walls; D. Field image of living, dead and differentiated cells after specific CFW staining of the cellulose micro fibrils in the cell walls; E. Differentiating cells representing intermediate state - combined staining with FDA (visible ruptured vacuole) and PI staining of a nucleus in dead cells; F. PI staining of a hollow differentiating cell with preserved nuclei and lysed cell content; G. CFW staining of the secondary cell walls in a cluster of differentiated cells; H. Cell cluster consisting of a differentiated and differentiating vacuolated cells. Scale bars (A, B, C, E, G, H) = 20 μm; D = 50 μm; F = 10 μm. Images were collected by using a TCS SP2 AOBS confocal laser scanning microscopy system (Leica-Microsystems GmbH, Mannheim, Germany) mounted on an inverted Leica DM IRE2 microscope. Three different lasers (405, 488 and 561 nm) were employed for excitation and three emission channels for fluorescence imaging and one separate channel for non-confocal transmission imaging. Overlays and orthogonal projections were made using the Leica Confocal software. The images were taken 120 h after administration of 1 mg/L NAA and 1 mg/L BA and stained with FDA (fluorescence visible in the cytoplasm of living cells and as faded green in the ruptured vacuole), PI (stains nuclei in dead cells) and CFW (specific for cell wall visualization). Cyt, cytoplasm; cw, cell wall; dc, dead cell; difc, differentiated cell; lc, living cell; L-TE, living TE; nu, nucleus; scw, secondary cell wall; v, vacuole.

**Figure 5:**
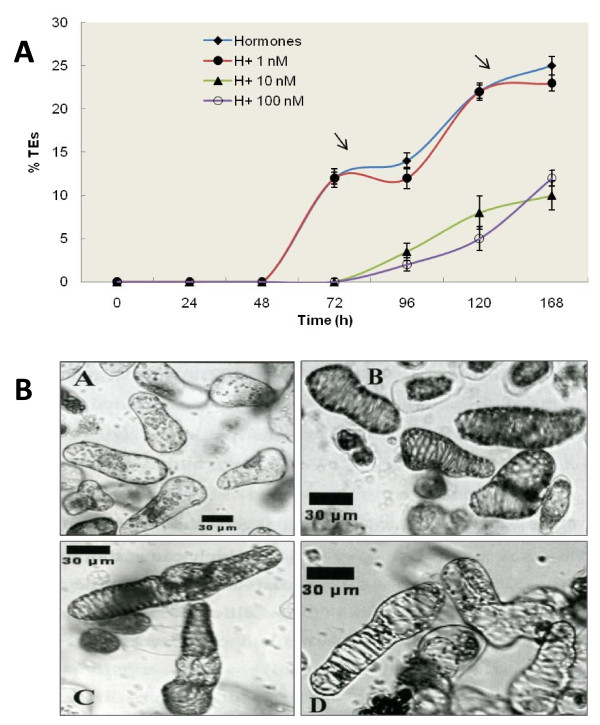
**Effect of Z-Asp-CH2-DCB on morphology, kinetics and yield of TEs generation and interference with the two 'waves' of TE differentiation in xylogenic Zinnia cell cultures**. A. Time course of TE formation; B. Morphology of formed TEs. TEs yield was calculated as a percentage of formed TEs to the initial number of living cells in 24 h intervals after addition of 1 mg/L NAA and 1 mg/L BA (hormones) and a combination of the hormones with 1, 10 and 100 nM of the irreversible broad spectrum caspase inhibitor Z-Asp-CH2-DCB (H+1 nM; H+10 nM; H+100 nM), until completion of TE differentiation. Error bars indicate SEM _(n-1)_. Image A. Non-induced control culture; Image B. Induced culture treated with 1 mg/L NAA and 1 mg/L BA; Image C. Hormones +10 nm of the irreversible broad spectrum caspase inhibitor Z-Asp-CH2-DCB; Image D. Hormones + 100 nM Z-Asp-CH2-DCB. The images were taken by differential interference contrast microscopy (Nikon DIC/Fluorescence microscope) using Image J software (Wayne Rasband, National Institute of Health, USA), 120 h after the addition of 1 mg/L NAA and 1 mg/L BA and a combination of the hormones with 10 and 100 nM Z-Asp-CH2-DCB. Scale bars = 30 μm.

Bright green fluorescence of FDA in the viable cells showed diffuse nuclei, intact cytoplasm and an intact vacuole, non permeable for the dye (Fig. 4A). A more evenly distributed and weaker green fluorescence was visible after vacuole rupture in the dead non-differentiated cells (Fig. 4B), presumably due to FDA influx into the ruptured vacuole. Double FDA-PI staining reveals a living cell with preserved cytoplasm and intact vacuole and a single SCW (Fig. 4B). The reason for using PI staining was that in addition to its ability to penetrate the nucleus in a dead cell, PI is also perfect for staining of cell walls, normally emitting red fluorescence. In fig. 4B, PI fluorescence is visualised in pink pseudocolor to distinguish from calcofluor staining of SCW (Fig. 4D, G, H) and to visualise the nuclei in the dead TEs. No "dead" nucleus appeared in the living TE. Fully differentiated TEs showed lignin auto fluorescence from the secondary cell walls (Fig. 4C). Auto fluorescence was first observed 6-12 h after vacuole rupture, suggesting that lignification occurs after cellulose deposition and vacuole collapse. CFW staining of the cellulose fiber in the secondary cell walls allowed qualitative analysis of the TE differentiation including distinguishing the different stages of the formation of cell wall thickenings as represented in the living (lacking secondary walls), differentiating (incomplete secondary cell walls) and fully differentiated (completed secondary wall patterning) vessel elements (Fig. 4D). The late stage of TE formation was preceded by vacuole rupture while the compacted and presumably non-functioning nucleus was still preserved and clearly distinguishable at combined staining with FDA (enters the ruptured vacuole) and PI (red colored nucleus; Fig. 4E and 4F). The maturation of TEs was completed by autolysis of the cell content followed by degradation of the compacted nucleus, to form a hollow differentiated cell (Fig. 4G). By CFW staining, aggregations of fused TEs with completed secondary cell wall patterning were observed (Fig. 4G). The image of a cluster consisting of a differentiated and differentiating cells showed that the deposition of secondary cell walls precedes the vacuole rupture (Fig. 4H). The morphology of the living and dead cells and of formed TEs was identical and the consecutive stages of TE formation appeared in the same order in cultures with and without addition of caspase inhibitors (Fig. 5), although TE formation was considerably delayed in the latter.

### Influence of the caspase inhibitor Z-Asp-CH2-DCB on the kinetics and yield of TEs

A concentration range of 1, 10 and 100 nM Z-Asp-CH2-DCB was used to test the ability of this caspase inhibitor to influence the timing of appearance and the yield of the TEs produced in the xylogenic Zinnia cell cultures. TEs were first detected after 48 h in hormone-treated cultures and in cultures treated with hormones and the lowest concentration (1 nM) of Z-Asp-CH2-DCB. A 24 h delay of the initiation of trans-differentiation was observed at higher inhibitor concentrations (Fig. 5A). The final percentage of produced TEs decreased with increasing concentration of the caspase inhibitor and, 168 h after the induction of trans-differentiation the TE yield in presence of 10 or 100 nM Z-Asp-CH2-DCB was approximately 40% of that in the non inhibitor-treated cells. Morphologically the formed TEs looked similar, irrespective of the treatments (Fig. 5B). No further TE development was observed at later time points (data not shown).

### Influence of the caspase inhibitor Z-Asp-CH2-DCB on the dimensions of produced TEs

**Figure 6:**
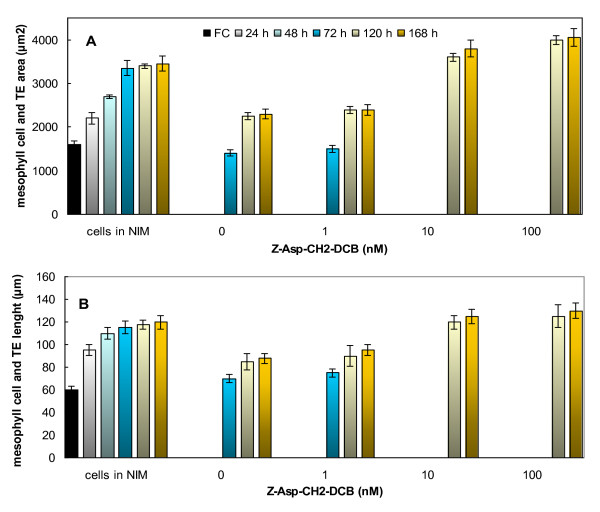
**Effect of Z-Asp-CH2-DCB on the dimensions of the TEs produced in Zinnia cell suspension cultures**. A. Effect of Z-Asp-CH2-DCB on TE area; B. Effect of Z-Asp-CH2-DCB on TE length. The dimensions were measured at consecutive time points of 24 and 48 h intervals until the completion of TE differentiation at 168 h after the treatments by using differential interference contrast microscopy (Nikon DIC/Fluorescence microscope) with Image J software (Wayne Rasband, National Institute of Health, USA). The cells were treated with 1 mg/L NAA and 1 mg/L BA (0) and combinations of the hormones with 1, 10 and 100 nM of the irreversible broad spectrum caspase inhibitor Z-Asp-CH2-DCB (1, 10 and 100). NIM -non-inductive media. Error bars indicate SEM _(n-1)_.

To study the effect of a caspase inhibitor on the dimensions of the TEs, the length and area of completed TEs were measured in hormone-induced cultures in the presence of hormones and 1, 10 or 100 nM Z-Asp-CH2-DCB. DIC microscopy revealed that proportional to the increase of Z-Asp-CH2-DCB concentration in the cultures, the length and area of the TEs increased (Fig. 6A, B). While the average area of the TEs produced in the non-inhibitor treated cultures was about 2400 μm^2^, a 40% larger area was measured of the TEs generated at the application of 100 nM Z-Asp-CH2-DCB (Fig. 6A). Similarly, the length of completed TEs was approximately 43% more in response to addition of the caspase inhibitor (Fig. 6B).

### Caspase inhibitor Z-Asp-CH2-DCB affects the observed two "waves" of TE differentiation

It was observed that the TE differentiation in the xylogenic Zinnia cultures often occurs in two "waves" (Fig. 5A); the first wave, resulted in smaller TEs than those produced in the second wave (Fig. 6A, B; compare cell size at 72 h (during first wave) with 120 h (during second wave). The administration of 10 or 100 nM Z-Asp-CH2-DCB led to only one wave (Fig. 5A) of TE trans-differentiation producing relatively large TEs (Fig. 6A, B). Both length and area of TEs formed in cultures treated with caspase inhibitor were about 30% larger than TEs formed during the second wave in non-inhibitor treated cultures.

## Discussion

### Human caspase inhibitors suppress TE formation in Zinnia cell cultures

Various signalling molecules have been associated with the regulation of xylogenesis [21,33,36,54-56], but still unresolved questions remain. One such intriguing question is whether the PCD during TE formation in Zinnia is indeed independent on caspase-like proteases as has been suggested by other authors [28,53]. Caspase-like proteases have been associated with apoptotic-like PCD such as PCD during plant-pathogen interactions and in response to toxic chemicals. The assumption that PCD during TE cell death is eventually a form of autophagy [57], may indeed suggest that caspase-like proteases are not of major importance. However, the exact category of PCD that occurs during TE differentiation in Zinnia has not yet been clearly defined.

The widespread opinion that caspase-like proteases do not participate in PCD during TE differentiation [28,53] is based on the observations that caspase inhibitors do not prevent TE formation, but to date no experimental details on the effects of caspase inhibitors have been published.

Applied caspase inhibitors to non hormone-induced cells increased cell viability; applied to hormone-induced cells they substantially reduced the number of formed TEs (Fig. 1A). As the TEs are formed through trans-differentiation of the living mesophyll cells, the abolishment of TE formation by caspase inhibitors suggests that they modulate the process of trans-differentiation. It is generally accepted that these inhibitors target plant proteases involved in cell death such as VPE and the proteasome [10-12,15-18,20,58]. Therefore, we may assume that also in Zinnia xylogenic cultures the effect of caspase inhibitors is associated with their effect on delaying cell death. However, we can not rule out that in xylogenic cultures other developmental processes, not related to PCD, may be affected by these inhibitors. Whether this is true also depends on the definition of PCD. According to most authors, PCD is the process that ultimately leads to death of the cell [2,4,5,16,22,23,26,31-33]. In this view PCD not only refers to the final event in cell death (rupture of the tonoplast) but also to the processes leading to this final event. It may even be argued that the trans-differentiation process leading to death of the cell may be regarded as a specific form of PCD that is accompanied by secondary wall thickening.

Considering this, it is hard to decide on whether caspase inhibitors suppress PCD or the developmental events involved in trans-differentiation as both processes may be intimately related. In addition, a different action of caspase inhibitors on the delay of TE development and on suppression eventual death of the cell may also be considered.

The asparaginyl-specific cysteine protease VPE that has been found as a key component of virus-induced plant PCD is structurally (although distantly) related to human caspases. VPE has been shown to express caspase-1 like activity; it plays a role in posttranslational modification of a variety of vacuolar proteases [17,18] and is a suggested component in the activation of proteins involved in vacuolar collapse during plant PCD. Here we show that caspase-1 inhibitor Ac-YVAD-CMK suppresses the TE differentiation, which suggests that VPE might participate in the vacuole-driven cellular dismantlement. However, this inhibitor had only a minor effect on TE development, it is hard to definitely assume a role of VPE in vacuole collapse during TE cell death (Fig. 1). Caspase-3 inhibitor Ac-DEVD-CHO has been found to block cell death in bacterial and chemical-induced cell death in plants [58]. Immunohistochemistry and immunoelectron microscopy revealed that caspase-3-like protease is distributed in the cytoplasm and the cell walls of developing tracheary elements in *Cucurbita moshata *and it was suggested to be involved in tonoplast rupture [52]. The suppression of TE formation, achieved after the addition of Ac-DEVD-CHO (Fig. 1A) in our experiments with Zinnia cells also indicates that caspase-3-like activity might be involved in TE PCD.

The prevention of cell death at the use of pan-caspase inhibitors is a way of simultaneously blocking the entire class of caspases and any caspase-related enzymes and provides an indication that the cell death is caspase-dependent. In addition to caspase 1 and caspase 3-specific inhibitors, the broad range caspase inhibitor Z-Asp-CH2-DCB also remarkably reduced the number of formed TEs. Plants do not have similar caspases as animal cells, but do show caspase-like activities (cleavage adjacent aspartate residues). Z-Asp-CH2-DCB is therefore a very suitable inhibitor as it also inhibits proteases with caspase-like activity of which we do not yet know the exact required sequence at the active site. We have previously shown that Z-Asp-CH2-DCB is a potent PCD suppressor in tomato suspension cells exposed to a variety of cell death inducers [11,59,60], as well as in mastoparan (wasp venom) treated *Chlamydomonas reinhardtii *(unpublished data). In addition, Z-Asp-CH2-DCB prevented cell death symptoms in *Alternaria alternata *AT toxin inoculated tobacco leaves [61]. Taken together, the effects of the applied caspase inhibitors on trans-differentiation in Zinnia cells suggest that their mode of action is through suppression of cell death, eventually resulting in delayed and suppressed TE formation.

Administration of the cysteine protease inhibitors IA and NEM in the cultures completely abolished the process of differentiation (data not shown); the cysteine protease inhibitor E64 substantially delayed the process. The inhibitory effect of these chemicals is in line with reports from other laboratories [46,62]. The complete prevention of differentiation, found as an effect of IA and NEM, might be due to inhibition of a broad range of cysteine proteases that participate in early stages of differentiation [46] or due to inhibition of extracellular proteases [29] that are released from differentiating cells or during the autolysis of dying cells and that may communicate with PCD-associated differentiation events in the remaining living cells. This idea is also reinforced by the effect of E64, being poorly cell permeable it most probably exerts its action mainly on extracellular cysteine proteases. At the administration of caspase inhibitors the trans-differentiation was delayed but not completely abolished in comparison to application of IA and NEM. The effect of caspase inhibitors is unlikely to be attributed to their effect on extra cellular proteases as their targets are considered intra cellular proteins. No information is available about a possible release and activity of caspase-like proteases in the extracellular space, comparable to what has been shown for other cysteine proteases involved in differentiation [29].

### Caspase inhibitors prevent DNA fragmentation during TE differentiation

Apoptotic PCD in animal cells is associated with a DNA laddering pattern resulting from inter nucleosomal DNA cleavage. In plant cells, DNA fragmentation into nucleosomal fragments of about 180 bp and multiples thereof has been demonstrated [11,22,63] but the occurrence of DNA ladders is not indispensable and has been shown depending on the nature and concentration of PCD inducers [64]. In hormone-induced differentiating Zinnia cell cultures we detected DNA breakdown reflected in a single band of 160-180 bp that was abolished in the presence of the broad range caspase inhibitor Z-Asp-CH2-DCB (Fig. 2). The reason that no "ladder" was observed is not clear. The portion of differentiated cells was always very small (approximately 20-25%) and at any specific time point only few cells may have been in the process of dying, which may have hindered detection of a "DNA ladder" on the gel. DNA breakdown was also confirmed by the appearance of TUNEL positive nuclei in the differentiating cells. TUNEL is a marker of free 3'-OH ends of DNA that accumulate at different kinds of cell death processes. TUNEL may not unequivocally discriminate between apoptotic-like inter nucleosomal DNA fragmentation and random DNA cleavage [65,66]. However, when used in conjunction with another confirmatory apoptotic-like PCD marker (e.g. DNA ladder, cell morphological features) it can be informative for identification of the cells in which PCD has actually taken place [66]. The dose-dependent decrease of the number of TUNEL positive nuclei in caspase inhibitor-treated cultures (Fig. 3) indicates a caspase-like protease-associated process of DNA degradation. In our experiments, apoptotic-like DNA fragmentation and TE cellular morphology (nuclear condensation, vacuole disruption) indicated the PCD nature of the process of differentiation for which the TUNEL results is supporting evidence. The correlation between the inhibition of TE formation and the inhibition of DNA fragmentation achieved by the caspase inhibitor is an additional indication that functional homologues of animal caspases may participate in the cell death events occurring during the process of TE differentiation in Zinnia cell cultures.

### Morphological characterization of TE differentiation

The morphological changes in differentiating Zinnia cells as observed by confocal laser-scanning microscopy confirmed the existence of the earlier reported consecutive stages in TE formation (Fig. 4) [28,31-34,51,67]. In addition, we also observed cells showing intermediate phases such as cells already occupied by a large vacuole with incomplete cellulosic secondary cell wall thickenings and cells with a ruptured tonoplast that nevertheless showed some staining with FDA (Fig. 4). The latter phenomenon was interpreted as a movement of FDA into the vacuole after tonoplast rupture.

Presented images (Fig. 4) show the typical mesophyll and TE cells morphology. What we demonstrate here is that in comparison to only hormone treated cultures, the order of events and the morphological features of trans-differentiation were not affected in the cell cultures treated with different caspase inhibitors. The caspase inhibitors, however caused significant delay of the onset of trans-differentiation including later appearance of CFW stained cellulose bands and shift of the timing for vacuolar collapse and autolysis of differentiating TEs.

### Caspase inhibitors delay the time of TE formation and promote formation of larger TEs

In a dose dependent manner, the caspase inhibitors lengthened the period for the occurrence of the individual trans-differentiation events leading to overall reduced amount of formed TEs (Fig. 5). Similar results were observed with other inhibitors and also in cultures of the Zinnia cultivar Purple Prince (data not shown). The observation that the caspase inhibitors did not re-order the TE trans-differentiation events indicates that these peptides delay the trans-differentiation process without disturbing the overall process of xylogenesis in Zinnia cells.

Dimensions of the xylem vessels, especially the length and diameter, greatly influence the water status of the plant. Manipulation of TE dimensions by e.g. environmental conditions affects the plant's water status and may influence agronomical characteristics [68-70]. Depending on the prevailing environmental conditions, plants are able to meet their water needs by adjusting the dimensions of their xylem vessels for better efficiency in conducting water and preventing possible vessel dysfunction that can happen e.g. due to air embolism [71-73]. It has earlier been reported that the suppression of mesophyll cell expansion by modulation of extracellular osmolarity during *in vitro *TE differentiation leads to formation of TE's of different sizes [74]. As PCD is an integral part of the TE differentiation process, we investigated if manipulation of PCD by a caspase inhibitor can change the final dimensions of TEs during trans-differentiation of Zinnia mesophyll cells. Indeed, we have observed larger TEs with delayed kinetics of formation in presence of the caspase inhibitors (Fig. 5, 6). This implicates that TE formation and their dimensions can be manipulated using caspase inhibitors. The *in vitro *Zinnia cell culture is a less complicated system than the whole plant, which makes it difficult to translate the results to the whole plant level. Methods to induce similar modification in xylem cells *in planta *might be a useful tool for altering the hydraulic properties of the xylem system and water relations of the plant.

### Caspase inhibitor modifies the two "waves' of TE formation

The trans-differentiation of mesophyll cells into TE's in Zinnia cultures usually occurs with two distinct waves. The first wave or "primary TE differentiation" involves differentiation that results in smaller average TE size as compared to the "secondary TE differentiation" (Fig. 6A, B). Also *in planta *it has been observed that secondary xylem TEs are larger than the primary xylem TEs [75]. Application of the caspase inhibitors eliminated the first wave of TE formation (Fig. 5) and yielded TE's that were larger than in the hormone only-treated cultures. (Fig. 6A, B). It is likely that cells undergoing differentiation secrete signaling compounds into the medium that repress the xylogenesis process in the other potentially differentiating cells. These other cells are delayed in entering the final irreversible stages of xylogenesis resulting in highly expanded cells and ultimately larger TE's. It is currently not known which regulatory molecules may be produced from the first wave of TE differentiation. The presence of the caspase inhibitors apparently mimicked the roles of the activities of the first wave of TE differentiation.

## Conclusions

In this study we have established that three human caspase inhibitors (Ac-YVAD-CMK, Ac-DEVD-CHO and Z-Asp-CH2-DCB) are potent suppressors of TE formation in xylogenic Zinnia cell cultures. Caspase inhibitors efficiently delayed the timing of TE generation and stimulated the development of TEs with larger dimensions (length and area), simultaneously modulating the usual 'two-wave' to "one-wave" pattern of TE production. Caspase inhibitors significantly inhibited the appearance of presumed PCD hallmarks such as DNA fragmentation as evidenced by agarose gel electrophoresis and appearance of TUNEL stained nuclei. The morphological observations of cellular structure of trans-differentiating TEs revealed structural changes involving condensation of the nucleus and cytoplasm, deposition of secondary cell walls, formation of a large vacuole, digestion of cellular content and disappearance of the nucleus and finally the formation of hollow differentiated TEs. This sequence of events was delayed, but not changed by the application of caspase inhibitors to the xylogenic cultures. Living TEs with SCW and intact vacuole were found both in caspase inhibitor-treated and in only hormone-induced cultures. Based on the obtained data we conclude that plant proteases with functional similarity to animal caspases participate in TE formation, presumably through their effect on PCD. Inhibition of caspase-like activities using caspase inhibitors or molecular genetic strategies might be promising tools for the regulation of xylogenesis *in planta*, although the surrounding tissues may pose a restriction on dimensions of TEs. Detailed studies involving detection of caspase-like enzyme activity, gene expression analysis and possibly the use of mutant plants will provide better understanding of the role of caspase-like dependent proteolysis in Zinnia TE cell death.

## Methods

### Cell cultures isolation and chemical treatments

Seedlings of Zinnia (*Zinnia elegans*) cv. Envy (Muller Bloemzaden BV, Lisse, Netherlands), were raised in peat-based commercial potting compost (Lentse Potgrond nr. 4; 85% peat, 15% clay, Lentse Potgrond, Lent, The Netherlands) at growing conditions of a 16 h day photoperiod; day/night temperature 25/20°C and lower than 70% RH. Mesophyll cells of Zinnia were isolated under sterile conditions from the first true leaves (3^rd ^leaf pair just emerging) of 14-day old seedlings, by gentle mechanical homogenization of surface-sterilized leaves in a cultures medium following the protocol of Fukuda and Komamine [30] with modifications for increasing the yield of produced TEs [33]. Briefly, the leaves were sterilized 10 min in 0.15% sodium hypochlorite containing 0.01% Triton X-100. Leaf homogenate was filtered through 50 μm nylon mesh and the filtrate was centrifuged at 150 g for 90 sec. The pellet was re-suspended with medium to achieve a cell density of 2.10^5 ^cells/ml (cell density measured with a haemocytometer) and the obtained suspension was cultured in the dark at 26°C in an orbital shaker at 80 rpm. Cell viability was determined with FDA and scored as percent of living cells to the total number of cells. Freshly isolated cultures contained more than 80% viable cells. For induction of TE differentiation, 48 h after the isolation, 3 ml portions of the cell cultures were treated with 1 mg/l α-naphthalene-acetic acid (NAA) and 1 mg/l benzyl aminopurine (BA) and cultured in sterile 6-well cultures plates at the above conditions, as described by Twumasi et al. [34]. Non-induced cultures (no hormones added) served as a negative control. Cell counting was executed by using Axiovert Carl Zeiss microscope at 100× magnification. The number of formed TEs (dead or living cells with secondary cell wall banding) was expressed as % TEs calculated on the basis of the % of living cells at the start of the experiment as only the cells that are initially alive can trans-differentiate [34].

For elucidating the involvement of caspase-like proteases in TE differentiation, the cell cultures were simultaneously treated with hormones and a range of concentrations of the irreversible broad-ranged human caspase-3 inhibitor benzyoxycarbonyl-Asp-2,6-dichlorobenzoyloxymethylketone (Z-Asp-CH2-DCB), the irreversible caspase-1 inhibitor Tyr-Val-ala-Asp-chloromethylketone (Ac-YVAD-CMK), and the reversible caspase-3 inhibitor Acyl-Asp-Glu-Val-l-aspartic acid aldehyde (Ac-DEVD-CHO). In addition, the cysteine protease inhibitors E64, IA and NEM were tested. Single application of the inhibitors to non-hormone-treated cells was tested to verify the possible toxicity of used chemicals. Controls with hormones and without inhibitors were also implied. DMSO was used as solvent for the drugs and the final concentration in the cultures did not exceed 0.1%, a concentration that appeared non-toxic for the cells.

### Detection of DNA fragmentation

Isolation and separation of DNA from the Zinnia cultured cells was performed essentially as described by de Jong et al. [11]. The cells were pelleted by centrifugation, frozen and grinded in liquid nitrogen. The resulting powder was mixed with 15 ml of 65°C extraction buffer (0.1 M Tris, 50 mM EDTA, 50 mM NaCl), freshly supplemented with 10 mM β-mercaptoethanol and 1 ml of 20% SDS, mixed thoroughly and, incubated at 65°C for 20 min. Then 5 ml of 5 M K-acetate was added to the mixture, this was kept on ice for 30 min and centrifuged for 30 min at 4°C, 3500 rpm. The supernatant was filtered through a tissue and collected in a clean tube, mixed with equal volume of isopropanol and immediately spun down for 5 min at 4°C. The pellet was dried and dissolved in 300 μl CTAB buffer (0.2 M Tris pH 7.5, 50 mM EDTA, 2 M NaCl, 2% cetyl-N, N, N triethyl ammonium bromide). Samples were incubated for 15 min at 65°C and subsequently extracted with an equal volume of chloroform. The water phase was precipitated with one volume of isopropanol and centrifuged for 5 min at 4°C. Finally, the pellet was dried and dissolved in 15 μl TE buffer (10 mM Tris pH 8.0, 1 mM EDTA), 0.1 μg μl^-1 ^DNAse-free RNase was added and the samples were incubated for 10 minutes at 37°C. Agarose gel (1.8% agarose) electrophoresis was performed with 15 μg DNA per lane.

### TUNEL assay

For *in situ *detection of DNA fragmentation in the cells undergoing xylogenesis, samples were fixed in microtubule stabilizing buffer (MSB, 100 mM PIPES (Piperazine-N, N'-bis(2-ethanesulfonic acid), 2.5 mM EGTA, 2.5 mM MgSO_4_.7 H2O, pH 7.4), containing 4% para formaldehyde (PFA) and 0.025% glutaraldehyde (GA), for 1 h at room temperature. The cells were immobilized on glass slides coated with poly-L-lysine and dried at room temperature. The fixed and dried samples were incubated for 20 min at 37°C in a permeabilization solution containing proteinase K (20 μg/ml proteinase K in 50 nM Tris-HCl, pH 7.5, 1 mg BSA). The immobilized cells were washed twice in phosphate-buffered saline (PBS, 200 mM NaCl, 50 mM Na_2_HPO_4, _50 mM NaH_2_PO_4_) and air dried [11]. Subsequently the cells were subjected to labelling of the 3'OH groups of nuclear DNA by TUNEL (Terminal deoxynucleotidyl transferase-mediated dUTP Nick End Labeling) assay using dioxigenin-dUTP according to the manufacturer's instructions (TUNEL kit, Roche Applied Science, Germany). TUNEL labelling was done by applying 50 μl of TUNEL reaction solution and incubating the slides in a humid atmosphere for 1 h at 37°C in the dark. The labelled samples were immersed in fresh MSB buffer (pH 6.9) for immediate observation or embedded in 99% glycerol antifade for later observation with Nikon fluorescent microscope at 519 nm emission wavelength. The number of TUNEL positive nuclei was scored as percent of cells with secondary wall thickening.

### Measurements of TE dimensions

Length and area of formed TEs were measured at 24 h and 48 h intervals after the induction of differentiation by using DIC microscopy (Nikon DIC/Fluorescence microscope). Image processing and measurements were done by Image J software (Wayne Rasband, National Institute of Health, USA). The dimensions were measured until completion of the process of differentiation in hormone and caspase inhibitor-treated cultures and at the same intervals in non-induced cell culture (NIM).

### Microscopic observations of cell morphology and imaging

The living cells were detected by FDA staining (green fluorescence visible in the living cells only) and the dead cells were distinguished by propidium iodide (PI) that visualizes nuclei in dead cells. Calcofluor white (0.0005%) was used to stain the patterns of cellulose bands profiling the secondary cell wall thickenings in the differentiated TEs. Images were collected by using a TCS SP2 AOBS confocal laser scanning microscopy system (Leica-Microsystems GmbH, Mannheim, Germany) mounted on an inverted Leica DM IRE2 microscope. Three different lasers (405, 488 and 561 nm) were employed for excitation and three emission channels for fluorescence imaging and one separate channel for non-confocal transmission imaging. The supplemental transmission imaging allowed the judgement of fluorescing and non-fluorescing cells, showed the boundary of all cells, also in a larger depth of field and, showed how many separate cells are present in a view. Overlays and orthogonal projections were made using the Leica Confocal software.

### Data analysis

Cell counting was performed in three non-overlapping randomly chosen microscopic fields per each sample. Presented data on cell viability and % TEs are averaged of at least three independent experiments after counting of approximately 80 cells per microscopic field. The dimensions (length and area) of the TEs are the average of at least 100 TEs. Values are compared by standard error of the means (SEM _(n-1)_).

## List of abbreviations

(AC-DEVD-CHO): acyl-Asp-Glu-Val-l-aspartic acid aldehyde; (AC-YVAD-CMK): acyl-Tyr-Val-Ala-Asp- chloromethylketone; (Z-ASP-CH2-DCB): benzyloxycarbonyl-Asp-2.6-dichlorobenzoyloxymethylketone; (BSA): bovine serum albumin; (CFW): calcofluor white; (FDA): fluorescein diacetate; (EDTA): diaminoethane tetraacetic acid; (DIC): differential interference contrast microscopy; (IA): iodoacetamide; (E-64): L-transepoxysuccinyl-leucylamido-[4-guanidino]butane; (NAA): α-naphthalene-acetic acid; (BA): N^6^benzylaminopurine; (NEM): N-ethylmaleimide; (PCD): programmed cell death; (PI): propidium iodide; (TUNEL): terminal deoxynucleotidyl transferase-mediated dUTP nick end labeling.

## Authors' contributions

PT and ETI shared their participation in the experimental design of the study, pharmacological analysis, light, fluorescent, confocal and DIC microscopic observations and measurements, DNA analysis, data processing and manuscript drafting. ETI also contributed to manuscript revision. TQ participated in the experimental work as a MSc thesis student. WvI and JHNS acted as daily supervisors of the experimental work. AMCE and OvK were involved in the coordination of the research program on xylogenesis and plant quality, OvK as chair of the Department Horticultural Production Chains and AMCE as chair of the Department of Plant Cell Biology. Both participated in the progress meetings.

EJW conceived and co-ordinated the study on PCD in xylogenic Zinnia cultures and drafted and revised the final version of the manuscript. All authors read and approved the final manuscript.

## Author's information

PT was employed as a PhD student at Wageningen University, Department Horticultural Production Chains from 2003 to 2007 and successfully defended his PhD Thesis "Hydraulic properties of *Zinnia elegans*: from cellular development *in vitro *to performance *in planta*" of which this study is a part. He is currently appointed as Lecturer at the Department of Biochemistry and Biotechnology, Kwame Nkrumah University of Science and Technology (KNUST), Kumasi, Ghana. ETI is qualified in plant physiology with a focus on programmed cell death, cell cultures, phyto hormones, stress and post-harvest physiology. In this study she participated through 21 months EC FP6 Marie Curie Intra-European Fellowship project "Regulating plant quality by controlling xylem vessel dimensions during xylogenesis" at Wageningen University, Agrotechnology and Food Science Group (AFSG), Wageningen, The Netherlands. At present ETI occupies a position of Associate Professor at the Institute of Ornamental Plants, Sofia, Bulgaria. TQ participated in the work when she was a MSc student, currently she is a PhD student at Wageningen University. WvI is Assistant Professor at Wageningen University, Department Horticultural Production Chains, his expertise is, among others, in effects of environmental conditions on plant growth and product quality and is an expert in xylem vessel functioning and its relation to quality of detached plant parts. JHNS is Associate Professor in the Laboratory of Plant Cell Biology, with a main expertise in structural plant cell biology and embryogenesis. AMCE is Professor of Plant Cell Biology of Wageningen University with main expertise in cytoskeleton and cell wall formation. OvK is chair holder of the Department Horticultural Production Chains of Wageningen University with main expertise in chain management and non-destructive measurements of processes related to product quality. EJW is Professor in the Department Horticultural Production Chains and project manager at the Wageningen University Research Institute "Food and Biobased Research". EJW has a broad background in (postharvest) plant biology and has long term experience in plant programmed cell death research using, among others, different types of cell cultures.

## References

[B1] Kerr JF Wyllie AH Currie AR Apoptosis: a basic biological phenomenon with wide ranging implications in tissue kinetics British J Cancer 1972 26 239 257 10.1038/bjc.1972.33 PMC2008650 4561027

[B2] Pennell RI Lamb C Programmed cell death in plants Plant Cell 1997 9 1157 1168 10.1105/tpc.9.7.1157 12237381 PMC156988

[B3] Heath MC Hypersensitive response-related death Plant Mol Biol 2000 44 323 334 10.1023/A:1026592509060 11199391

[B4] Fukuda H Programmed cell death of tracheary elements as a paradigm in plants Plant Mol Biol 2000 44 245 253 10.1023/A:1026532223173 11199386

[B5] Lam E Kato N Lawton M Programmed cell death, mitochondria and the plant hypersensitive response Nature 2001 411 848 853 10.1038/35081184 11459068

[B6] van Doorn WG Woltering EJ Many ways to exit? Cell death categories in plants Trends Plant Sci 2005 10 117 122 10.1016/j.tplants.2005.08.003 15749469

[B7] Steller H Mechanisms and genes of cellular suicide Science 1995 267 1445 1449 10.1126/science.7878463 7878463

[B8] Hengartner MO The biochemistry of apoptosis Nature 2000 407 770 776 10.1038/35037710 11048727

[B9] Grütter MG Caspases: key players in programmed cell death Curr Opin Struct Biol 2000 10 649 655 10.1016/S0959-440X(00)00146-9 11114501

[B10] Del Pozo O Lam E Caspases and programmed cell death in the hypersensitive response of plants to pathogens Curr Biol 1998 8 1129 1132 10.1016/S0960-9822(98)70469-5 9778530

[B11] De Jong AJ Hoeberichts FA Yakimova ET Maximova E Woltering EJ Chemical-induced apoptotic cell death in tomato cells: involvement of caspase-like proteases Planta 2000 211 656 662 10.1007/s004250000341 11089678

[B12] Elbaz M Avni A Weil M Constitutive caspase-like machinery executes programmed cell death in plant cells Cell Death Differ 2002 9 726 733 10.1038/sj.cdd.4401030 12058273

[B13] Woltering EJ van der Bent A Hoeberichts FA Do plant caspases exist? Plant Physiol 2002 130 1764 1769 10.1104/pp.006338 12481059 PMC1540272

[B14] Chichkova NV Kim SH Titova ES Kalkum M Morozov VS Rubtsov YP Kalinina NO Taliansky ME Vartapetian AB A plant caspase-like protease activated during the hypersensitive response Plant Cell 2004 16 157 171 10.1105/tpc.017889 14660804 PMC301402

[B15] Woltering EJ Death proteases come alive Trends Plant Sci 2004 9 469 472 10.1016/j.tplants.2004.08.001 15465679

[B16] Coffeen WC Wolpert TJ Purification and characterisation of serine proteases that exhibit caspase-like activity and are associated with programmed cell death in *Avena sativa* Plant Cell 2004 16 857 873 10.1105/tpc.017947 15020745 PMC412861

[B17] Hatsugai N Kuroyanagi M Yamada K Meshi T Tsuda S Kondo M Nishimura M Hara Nishimura A plant vacuolar protease, VPE, mediates virus-induced hypersensitive cell death Science 2004 305 855 858 10.1126/science.1099859 15297671

[B18] Rojo E Martin R Carter C Zouhar J Pan S Plotnikova J Jin H Paneque M Sanchez-Serrano JJ Baker B Ausubel FM Raikhel NV VPE gamma exhibits a caspase-like activity that contributes to defense against pathogens Curr Biol 2004 14 1897 1906 10.1016/j.cub.2004.09.056 15530390

[B19] Bozhkov PV Filonova LH Suarez MF Programmed cell death in plant embryogenesis Curr Top Dev Biol 2005 67 135 179 10.1016/S0070-2153(05)67004-4 15949533

[B20] Rotari VI He R Gallois P Death by proteases in plants: whodunit Physiol Plant 2005 123 376 385 10.1111/j.1399-3054.2005.00465.x

[B21] Bonneau L Ge Y Drury GE Gallois P What happened to plant caspases? J Exp Bot 2008 59 491 499 10.1093/jxb/erm352 18272922

[B22] Wang H Juan LI Bostock RM Gilchrist DG Apoptosis: a functional paradigm of programmed cell death induced by a host selective phytotoxin and invoked during development Plant Cell 1996 8 375:391 12239387 10.1105/tpc.8.3.375 PMC161107

[B23] Groover A DeWitt N Heidel A Jones A Programmed cell death of plant tracheary elements differentiating *in vitro* Protoplasma 1997 196 197 211 10.1007/BF01279568

[B24] Sanmartin M Jaroszewski L Raikhel NV Rojo E Caspases. Regulating death since the origin of life Plant Physiol 2005 137 841 847 10.1104/pp.104.058552 15761210 PMC1065385

[B25] Gladish DK Xu JP Niki T Apoptosis-like programmed cell death occurs in procambium and ground meristem of pea (*Pisum sativum*) root tips exposed to sudden flooding Ann Bot 2006 97 895 902 10.1093/aob/mcl040 16533830 PMC2803422

[B26] Love AJ Milner JJ Sadanandom A Timing is everything: regulatory overlap in plant cell death Trends Plant Sci 2008 13 589 595 10.1016/j.tplants.2008.08.006 18824399

[B27] Geitmann A Franklin-Tong VE Emons AC The self-incompatibility response in *Papaver rhoeas *pollen causes early and striking alterations to organelles Cell Death Diff 2004 11 812 822 10.1038/sj.cdd.4401424 15044967

[B28] Fukuda H Tracheary element differentiation Plant Cell 1997 9 1147 1156 10.1105/tpc.9.7.1147 12237380 PMC156987

[B29] Groover A Jones AM Tracheary element differentiation uses a novel mechanism coordinating programmed cell death and secondary cell wall synthesis Plant Physiol 1999 119 375 384 10.1104/pp.119.2.375 9952432 PMC32113

[B30] Fukuda H Komamine A Establishment of an experimental system for the study of tracheary element differentiation from single cells isolated from the mesophyll of *Zinnia elegans* Plant Physiol 1980 65 57 60 10.1104/pp.65.1.57 16661142 PMC440265

[B31] Fukuda H Xylogenesis: initiation, progression and cell death Annu Rev Plant Physiol Plant Mol Biol 1996 47 299 325 10.1146/annurev.arplant.47.1.299 15012291

[B32] Roberts K McCann MC Xylogenesis: the birth of a corpse Curr Opin Plant Biol 2000 3 517 522 10.1016/S1369-5266(00)00122-9 11074384

[B33] Turner S Gallois P Brown D Tracheary element differentiation Annu Rev Plant Biol 2007 58 407 433 10.1146/annurev.arplant.57.032905.105236 17472568

[B34] Twumasi P Schel JHN van Ieperen W Woltering E Van Kooten O Emons AMC Establishing *in vitro *Zinnia elegans cell suspension cultures with high tracheary element differentiation Cell Biol Intern 2009 33 523 533 10.1016/j.cellbi.2009.01.019 19232395

[B35] Aloni R The role of cytokinin in organized differentiation of vascular tissues Aust J Plant Physiol 1993 20 601 608 10.1071/PP9930601

[B36] Fukuda H Signals that control plant vascular cell differentiation Nat Rev Mol Cell Biol 2004 5 379 391 10.1038/nrm1364 15122351

[B37] Matsubayashi Y Takagi L Omura N Morita A Sakagami Y The endogenous sulfated pentapeptide phytosulfokine-alpha stimulates tracheary element differentiation of isolated mesophyll cells of Zinnia Plant Physiol 1999 120 1043 1048 10.1104/pp.120.4.1043 10444087 PMC59337

[B38] Kuriyama H Fukuda H Regulation of tracheary element differentiation J Plant Growth Regul 2001 20 35 51 10.1007/s003440010006

[B39] Gabaldon C Ros LVG Pedreno MA Barcelo AR Nitric oxide production by the differentiating xylem of *Zinnia elegans* New Phytol 2005 165 121 130 10.1111/j.1469-8137.2004.01230.x 15720627

[B40] Dengler NG Regulation of vascular development J Plant Growth Regul 2001 20 1 13 10.1007/s003440010008

[B41] Motose H Iwamoto K Endo S Demura T Sakagami Y Matsubayashi Y Moore KL Fukuda H Involvement of phytosulfokine in the attenuation of stress response during the transdifferentiation of Zinnia mesophyll cells into tracheary elements Plant Physiol 2009 150 437 447 10.1104/pp.109.135954 19270060 PMC2675742

[B42] Hosokawa M Suzuki S Umezawa T Sato Y Progress of lignification mediated by intercellular transportation of monolignols during tracheary element differentiation of isolated Zinnia mesophyll cells Plant Cell Physiol 2001 42 959 968 10.1093/pcp/pce124 11577190

[B43] Tokunaga N Sakakibara N Umezawa T Ito Y Fukuda H Sato Y Involvement of extracellular dilignols in lignification during tracheary element differentiation of isolated Zinnia mesophyll cells Plant Cell Physiol 2005 46 224 232 10.1093/pcp/pci017 15659440

[B44] Motose H Sugiyama M Fukuda H A proteoglycan mediates inductive interaction during plant vascular development Nature 2004 429 873 878 10.1038/nature02613 15215864

[B45] Beers EP Freeman TB Proteinase activity during tracheary element differentiation in Zinnia mesophyll cultures Plant Physiol 1997 113 873 880 12223649 10.1104/pp.113.3.873 PMC158207

[B46] Ye Z-H Varner JE Induction of cysteine and serine proteases during xylogenesis in *Zinnia elegans* Plant Mol Biol 1996 30 1233 1246 10.1007/BF00019555 8704132

[B47] Kubo M Udagawa M Nishikubo N Horiguchi G Yamaguchi M Ito J Mimura T Fukuda H Demura T Transcription switches for protoxylem and metaxylem vessel formation Genes Dev 2005 19 1855 1860 10.1101/gad.1331305 16103214 PMC1186185

[B48] Aoyagi S Sugiyama M Fukuda H BEN1 and ZEN1 cDNAs encoding S1-type DNases that are associated with programmed cell death in plants FEBS Lett 1998 429 134 138 10.1016/S0014-5793(98)00563-8 9650576

[B49] Ito J Fukuda H ZEN1 is a key enzyme in degradation of nuclear DNA during programmed cell death of tracheary elements Plant Cell 2002 14 3201 3211 10.1105/tpc.006411 12468737 PMC151212

[B50] Avci U Petzold HE Ismail IO Beers EP Haigler CH Cysteine proteases XCP1 and XCP2 aid micro-autolysis within the intact central vacuole during xylogenesis in Arabidopsis roots Plant J 2008 56 303 315 10.1111/j.1365-313X.2008.03592.x 18573193

[B51] Obara K Kuriyama H Fukuda H Direct evidence of active and rapid nuclear degradation triggered by vacuole rupture during programmed cell death in *Zinnia* Plant Physiol 2001 125 615 626 10.1104/pp.125.2.615 11161019 PMC64863

[B52] Hao X Qian J Xu S Son X Zhu J Location of caspase 3-like protease in the development of sieve element and tracheary element of stem in *Cucurbita moschata* J Integrative Plant Biol 2008 50 1499 1507 10.1111/j.1744-7909.2008.00719.x 19093968

[B53] McCann MC Stacey NJ Roberts K Targeted cell death in xylogenesis Programmed Cell Death in Animals and Plants 2000 Oxford: Scientific Publishers Ltd 193 201 12090008

[B54] Demura T Fukuda H Novel vascular cell-specific genes whose expression is regulated temporarily and spatially during vascular system development Plant Cell 1994 6 967 981 10.1105/tpc.6.7.967 8069107 PMC160493

[B55] Milioni D Sado PE Stacey NJ Roberts K McCann MC Early gene expression associated with the commitment and differentiation of a plant tracheary element is revealed by cDNA-amplified fragment length polymorphism analysis Plant Cell 2002 14 2813 2824 10.1105/tpc.005231 12417703 PMC152729

[B56] Jung JH Kim SG Seo PJ Park CM Molecular Mechanisms Underlying Vascular Development Advances in Botanical Research Incorporating Advances in Plant Pathology 2008 48 London: Academic Press Ltd 1 68

[B57] Weir IE Maddumage R Allan AC Ferguson IB Flow cytometric analysis of tracheary element differentiation in *Zinnia elegans *cells Cytometry Part A 2005 68 A:81 91 10.1002/cyto.a.20194 16228979

[B58] Hatsugai N Iwasaki S Tamura K Kondo M Fuji K Ogasawara K Nishimura M Hara-Nishimura I A novel membrane fusion-mediated plant immunity against bacterial pathogens Genes Dev 2009 23 2496 2506 10.1101/gad.1825209 19833761 PMC2779742

[B59] Yakimova ET Kapchina-Toteva VM Laarhoven L-J Harren FM Woltering EJ Involvement of ethylene and lipid signalling in cadmium-induced programmed cell death in tomato suspension cells Plant Physiol Biochem 2006 44 581 589 10.1016/j.plaphy.2006.09.003 17079154

[B60] Yakimova ET Kapchina-Toteva VM Woltering EJ Signal transduction events in aluminum-induced cell death in tomato suspension cells J Plant Physiol 2007 164 702 708 10.1016/j.jplph.2006.03.018 16781012

[B61] Yakimova ET Yordanova ZP Slavov S Kapchina-Toteva VM Woltering EJ Biochemical events in *Alternaria alternata *AT toxin-induced cell death in tobacco J Phytopathol 2009 157 592 601 10.1111/j.1439-0434.2008.01535.x

[B62] Minami A Fukuda H Transient and specific expression of a cysteine endopeptidase associated with autolysis during differentiation of Zinnia mesophyll cell into tracheary elements Plant Cell Physiol 1995 36 1599 1606 8589934

[B63] McCabe PF Levine A Meijer P-J Tapon NA Pennel RI A programmed cell death pathway activated in carrot cells cultured at low cell density Plant J 1997 12 267 280 10.1046/j.1365-313X.1997.12020267.x

[B64] Kuthanova A Opatrny Z Fischer L Is internucleosomal DNA fragmentation an indicator of programmed death in plant cells? J Exp Bot 2008 59 2233 2240 10.1093/jxb/ern090 18436542 PMC2413271

[B65] Gavrieli Y Sherman Y Ben-Sasson SA Identification of programmed cell death *in situ *via specific labelling of nuclear DNA fragmentation J Cell Biol 1992 119 493 501 10.1083/jcb.119.3.493 1400587 PMC2289665

[B66] Wang M Hoekstra S van Bergen S Lamers GEM Oppedijk BJ van der Heijden MW de Priester W Schilperoort RA Apoptosis in developing anthers and the role of ABA in this process during androgenesis in *Hordeum vulgare *L Plant Mol Biol 1999 39 489 501 10.1023/A:1006198431596 10092177

[B67] Kuriyama H Loss of tonoplast integrity programmed in tracheary element differentiation Plant Physiol 1999 121 763 774 10.1104/pp.121.3.763 10557224 PMC59438

[B68] van Ieperen W Nijsse J Keijzer CJ van Meeteren U Induction of air embolism in xylem conduits of pre-defined diameter J Exp Bot 2001 52 981 991 10.1093/jexbot/52.358.981 11432915

[B69] van Ieperen W van Meeteren U Nijsse J Embolism repair in cut flower stems: a physical approach Postharvest Biol Tech 2002 25 1 4 10.1016/S0925-5214(01)00161-2

[B70] Twumasi P van Ieperen W Woltering EJ Emons AMC Schel JHN Schel JFH Van Meeteren U van Marwijk D Effects of water stress during growth on xylem anatomy, xylem functioning and vase life in three *Zinnia elegans *cultivars Acta Hort 2005 669 303 311

[B71] van Doorn WG Water relations of cut flowers Hort Rev 1997 18 1 85

[B72] Nijsse J van der Heijden GWAM van Ieperen W Keijzer CJ van Meeteren U Xylem hydraulic conductivity related to conduit dimensions along chrysanthemum stems J Exp Bot 2001 52 319 327 10.1093/jexbot/52.355.319 11283177

[B73] Tyree MT Plant hydraulics: the ascent of water Nature 2003 423 923 923 10.1038/423923a 12827177

[B74] Lee S Roberts AW Tracheary element differentiation is correlated with inhibition of cell expansion in xylogenic mesophyll suspension cultures Plant Physiol Bioch 2004 42 43 48 10.1016/j.plaphy.2003.10.005 15061083

[B75] Tuominen H Puech L Fink S Sundberg B A radial concentration gradient of indole -3-acetic acid is related to secondary xylem development in hybrid Aspen Plant Physiol 1997 115 577 585 12223825 10.1104/pp.115.2.577 PMC158517

